# Five-point scoring system based on clinicopathological data: A convenient criterion to determine prognosis of patients with colorectal carcinoma

**DOI:** 10.3892/ol.2013.1115

**Published:** 2013-01-08

**Authors:** TADAHIRO NOZOE, MAYUKO KOHNO, TOMOHIRO IGUCHI, TAKASHI MAEDA, TAKAHIRO EZAKI

**Affiliations:** Department of Surgery, Fukuoka Higashi Medical Center, Koga 811-3195, Japan

**Keywords:** colorectal carcinoma, histopathologic feature, five-point scoring system (FPSS)

## Abstract

The aim of this study was to elucidate the significance of a novel staging criterion, a five-point scoring system (FPSS), determined by five histopathological factors of colorectal carcinoma. These factors included depth of invasion, lymph node metastasis, lymphatic invasion, venous invasion and histopathological tumor type. In total, 357 patients with colorectal carcinoma who had had been treated by surgical resection were investigated. One point was assigned to each of the five aforementioned tumor-related pathological factors. The FPSS score was determined by an aggregate of the points. A significant difference was observed between the survival of patients with FPSS scores of 0 and 1, and 2 and 3 (P=0.0002); and FPSS scores of 2 and 3, and 4 and 5 (P<0.0001). We demonstrate that the FPSS is a convenient criterion for stratifying the prognosis of patients with colorectal carcinoma.

## Introduction

While there have been many criteria for determining the prognosis of patients with malignant tumors, such as the TNM staging criteria, these serve only to provide physicians with information regarding the outcome of patients during daily clinical analysis. In a previous study, we demonstrated that the Pathological Prognostic Score (PPS), determined based on histopatological data including depth of tumor, lymph node metastasis, lymphatic invasion and venous invasion, clearly classified the prognosis of patients with colorectal carcinoma (1).

Although undifferentiated carcinoma of the colon and rectum have been reputed to possess a more aggressive potential, which leads to a worse prognosis of patients (2), the criteria for determining the prognosis of patients with colorectal carcinoma reflected by this pathological type of tumor has not yet been presented. In this study, we investigated the significance of a novel staging criterion (a five-point scoring system; FPSS) for determining the prognosis of patients with colorectal carcinoma. The FPSS comprised five histopathological categories of patients with colorectal carcinoma: Depth of tumor, lymph node metastasis, lymphatic invasion, venous invasion and histopathological tumor type.

## Patients and methods

### Patients

In total, 357 patients with colorectal carcinoma, who had been treated by surgical resection at the Fukuoka Higashi Medical Center from January 1997 to January 2011, were evaluated. Forty-two patients had been treated with palliative resection due to the presence of distant metastasis and/or peritoneal dissemination. No patients had been treated with neoadjuvant therapy. Patients were aged between 24 and 91 years (mean, 69) and the group comprised 214 males and 143 females. The study was approved by the Ethics Committee of Fukuoka Higashi Medical Center, Koga, Japan. Written infomed consent was obtained from the patient.

### Pathological research

The clinicopathological factors were determined according to the general rules for clinical and pathological studies on cancer of the colon, rectum and anus, outlined by the Japanese Research Society for Cancer of the Colon and Rectum (3). Additionally, TNM tumor stages were determined by the TNM classification of malignant tumors prescribed by the International Union Against Cancer (4).

### FPSS

FPSS scores were determined by assigning one point to a more advanced result in each of the following categories: Tumor depth (T1 and 2 vs. T3 and 4); lymph node metastasis (positive vs. negative); lymphatic invasion (positive vs. negative); venous invasion (positive vs. negative) and histopathological tumor type (differentiated vs. undifferentiated tumor). Subsequently, FPSS scores were determined by an aggregate of points for each category and ranged from 0–5.

### Patient follow-up

Follow-up of patients was continued until mortality and only patients whose cause of death was colorectal carcinoma were included in the tumor-related deaths. The time period between surgery and death was termed the survival time.

### Statistical analysis

All statistical analyses were conducted using StatView version 5.0 (SAS Institute Inc, Cary, NC, USA). Then, a χ^2^ test was used to compare the difference in proportion values between FPSS scores. A Mann-Whitney U test was used to compare the mean ages of patients. Survival curves were conducted using the Kaplan-Meier method and a Mantel-Cox test was used to analyze their equality. P<0.05 was considered to indicate a statistically significant difference.

## Results

Each factor included in the FPSS (tumor depth, nodal metastasis, lymphatic invasion, venous invasion and histopathological tumor type) was found to be an indicator of worse prognosis in patients with colorectal carcinoma (Table I).

The study population was divided into three groups according to the FPSS score: 0 and 1 (153 patients, 42.9%); 2 and 3 (150 patients, 42.0%); and 4 and 5 (54 patients, 15.1%). A significant correlation was observed between FPSS score and the following tumor-related factors: Tumor depth, lymph node (nodal) metastasis, lymphatic invasion, venous invasion and proportion of curative resection (P<0.0001 for each factor; Table II).

**Figure 1:**
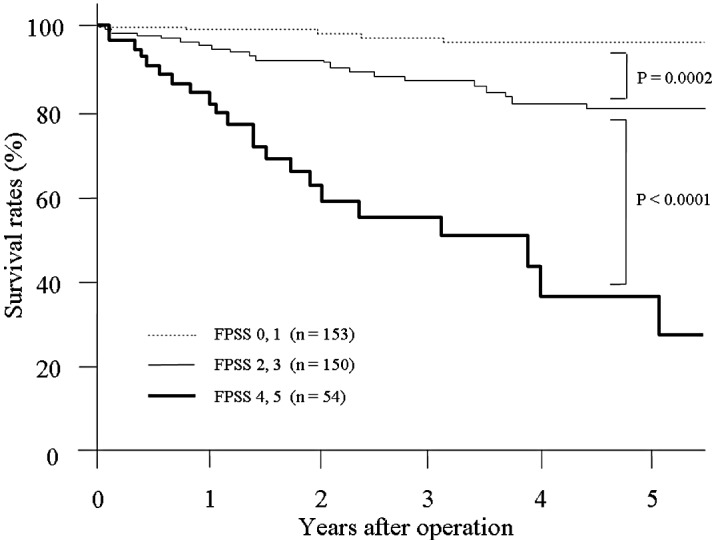
Survival curves. Dotted line, FPSS scores of 0 and 1; thin line, FPSS scores of 2 and 3; thick line, FPSS scores of 4 and 5. FPSS; five-point scoring system.

The 1-, 3- and 5-year survival rates of patients with FPSS scores of 0 and 1 were 99.3, 97.4 and 96.2%, respectively. The rates were 95.5, 87.1 and 80.5%, respectively, in patients with FPSS scores of 2 and 3; and 83.8, 54.7 and 35.8%, respectively, in patients with FPSS scores of 4 and 5. A statistically significant difference was observed between the survival of patients with FPSS scores of 0 and 1, and 2 and 3 (P= 0.0002); as well as FPSS scores of 2 and 3, and 4 and 5 (P<0.0001; Fig. 1).

**Figure 2:**
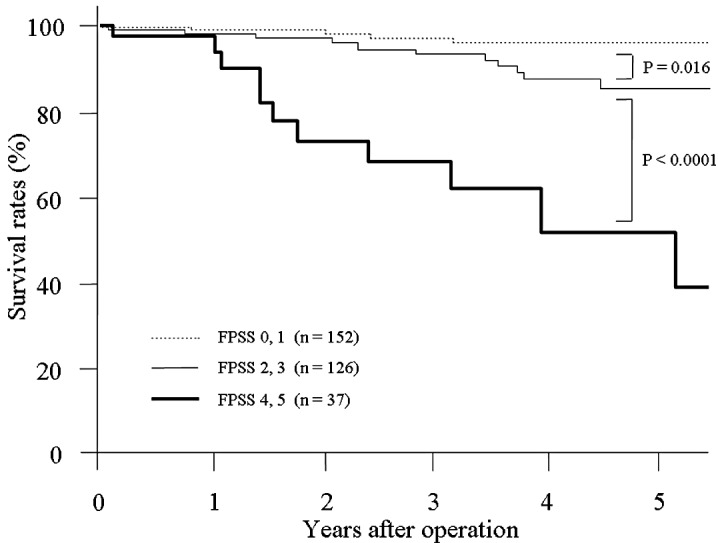
Survival curves for patients who had been treated with curative resection. Dotted line, FPSS scores of 0 and 1; thin line, FPSS scores of 2 and 3; thick line, FPSS scores of 4 and 5. FPSS; five-point scoring system.

Subsequently, an investigation restricted to 315 patients who had been treated with curative resection was performed. Similarly, a significant correlation was observed between FPSS scores and certain investigated tumor-related factors (Table III). The 1-, 3- and 5-year survival rates of patients with FPSS scores of 0 and 1 were 99.3, 97.4 and 96.2%, respectively. Such rates were 98.2, 93.6 and 85.8%, respectively, in patients with FPSS scores of 2 and 3; and 97.2, 67.7 and 51.3%, respectively, in patients with FPSS scores of 4 and 5. Additionally, a significant difference was observed between survival of patients with FPSS scores of 0 and 1, and 2 and 3 (P= 0.016); and FPSS scores of 2 and 3, and 4 and 5 (P<0.0001; Fig. 2).

## Discussion

We have previously demonstrated the prognostic significance of the Pathologic Prognostic Score (PPS), determined by pathological tumor-related factors including depth of the tumor, lymph node metastasis, lymphatic invasion and venous invasion, which has provided a useful prognostic stratification for patients with gastric carcinoma (5) and colorectal carcinoma (1).

Poorly differentiated or undifferentiated carcinoma, including poorly differentiated adenocarcinoma and mucinous carcinoma of the colon and rectum, has been reported to possess a more aggressive biological potential compared with differentiated carcinomas (2,6–9). Certain clinical and experimental studies have been conducted to identify the subtype among colorectal poorly differentiated carcinoma that has a more progressive potential or causes a more unfavorable prognosis of patients (10–13). However, to the best of our knowledge, there have been no studies regarding a criterion for determining the tumor stage of poorly differentiated or undifferentiated colon and rectal carcinoma.

Therefore, we set out to create an evolved criterion, FPSS, based on data regarding the histopathological tumor type, separating poorly differentiated and differentiated carcinoma, in addition to PPS, to potentially determine the prognosis of patients with colorectal carcinoma. A significant difference in prognosis was found between patients who had FPSS scores of 0 and 1, and 2 and 3; and FPSS scores of 2 and 3, and 4 and 5. Therefore, there is evidence to suggest that the quality of stratification observed in the classification system was useful. Moreover, an analysis of patients who had been treated with curative resection demonstrated similar results.

As emphasized in previous studies, a novel criterion for determining the prognosis of cancer patients has the potential for simple and useful application (1,5,14). While the five histopathological tumor-related factors comprising the FPSS are relatively common and the majority of medical institutes are capable of examining them, surgeons would benefit from the convenience of the clinical application of the FPSS in treating patients with colorectal carcinoma. This is due to the fact that the FPSS provides useful information regarding the clinical outcomes of patients. In conclusion, the FPSS may be a useful criteria for predicting the clinical outcome of patients with colorectal carcinoma.

## Figures and Tables

**Table I:** Analysis of survival rates based on five pathological factors.

Characteristic	No. of patients	5-year SR (%)	P-value
Depth of tumor			
T1 and T2	106	98.7	<0.0001
T3 and T4	251	74.5	
Nodal metastasis			
No	205	91.4	<0.0001
Yes	152	68.3	
Lymphatic invasion			
No	214	91.4	<0.0001
Yes	143	67.1	
Venous invasion			
No	259	88.5	<0.0001
Yes	98	63.2	
Histologic type			
Differentiated	325	86.3	<0.0001
Undifferentiated	32	37.5	

SR, survival rate.

**Table II:** Correlation between FPSS score and clinicopathological characteristics of patients.

	No. of patients (%)			
Characteristic	FPSS 0 and 1 (n=153)	FPSS 2 and 3 (n=150)	FPSS 4 and 5 (n=54)	P-value
Gender				
Male	95 (62.1)	92 (61.3)	27 (50.0)	0.267
Female	58 (37.9)	58 (38.7)	27 (50.0)	
Age (years ± SD)	69.6±10.7	69.8±10.9	68.8±12.1	0.906
Location of tumor				
Colon	108 (70.6)	106 (70.7)	38 (70.4)	0.999
Rectum	45 (29.4)	44 (29.3)	16 (29.6)	
Depth of tumor				
T1	46 (30.1)	0	0	<0.0001
T2	52 (34.0)	8 (5.3)	0	
T3	55 (35.9)	134 (89.4)	52 (96.3)	
T4	0	8 (5.3)	2 (3.7)	
Nodal metastasis				
No	147 (96.1)	55 (36.7)	3 (5.6)	<0.0001
Yes	6 (3.9)	95 (63.3)	51 (94.4)	
Lymphatic invasion				
No	146 (95.4)	67 (44.7)	1 (1.9)	<0.0001
Yes	7 (4.6)	83 (55.3)	53 (98.1)	
Venous invasion				
No	144 (94.1)	107 (71.3)	8 (14.8)	<0.0001
Yes	9 (5.9)	43 (28.7)	46 (85.2)	
Resection				
Curative	152 (99.3)	126 (84.0)	37 (68.5)	<0.0001
Non-curative	1 (0.7)	24 (16.0)	17 (31.5)	

FPSS, five-point scoring system.

**Table III:** Correlation between FPSS score and clinicopathological characteristics of patients treated with curative resection.

	No. of patients (%)			
Characteristic	FPSS 0 and 1 (n=152)	FPSS 2 and 3 (n=126)	FPSS 4 and 5(n=37)	P-value
Gender				
Male	95 (62.5)	79 (62.7)	19 (51.4)	0.419
Female	57 (37.5)	47 (37.3)	18 (48.6)	
Age (years ± SD)	69.6±10.8	70.2±11.0	71.8±10.9	0.268
Location of tumor				
Colon	108 (71.1)	92 (73.0)	24 (64.9)	0.630
Rectum	44 (28.9)	34 (27.0)	13 (35.1)	
Depth of tumor				
T1	46 (30.3)	0	0	<0.0001
T2	52 (34.2)	6 (4.8)	0	
T3	54 (35.5)	114 (90.4)	35 (94.6)	
T4	0	6 (4.8)	2 (5.4)	
Nodal metastasis				
No	146 (96.1)	48 (38.1)	3 (8.1)	<0.0001
Yes	6 (3.9)	78 (61.9)	34 (91.9)	
Lymphatic invasion				
No	145 (95.4)	56 (44.4)	1 (2.7)	<0.0001
Yes	7 (4.6)	70 (55.6)	36 (97.3)	
Venous invasion				
No	143 (94.1)	93 (73.8)	4 (10.8)	<0.0001
Yes	9 (5.9)	33 (26.2)	33 (89.2)	

FPSS, five-point scoring system.
